# Optimizing motion detection performance: Harnessing the power of squeeze and excitation modules

**DOI:** 10.1371/journal.pone.0308933

**Published:** 2024-08-19

**Authors:** Jabulani Brown Mpofu, Chenglong Li, Xinyan Gao, Xinxin Su

**Affiliations:** 1 School of Computer Science and Technology, Shandong Jianzhu University, Shandong, China; 2 Shandong Hoteam Software Co., Ltd., Jinan, China; University of Manitoba, CANADA

## Abstract

This paper introduces an innovative segmentation model that extends the U-Net architecture with a Squeeze and Excitation (SE) mechanism, designed to enhance the detection of moving objects in video streams. By integrating this model into the ViBe motion detection algorithm, we have significantly improved detection accuracy and reduced false positive rates. Our approach leverages adaptive techniques to increase the robustness of the segmentation model in complex scenarios, without requiring extensive manual parameter tuning. Despite the notable improvements, we recognize that further training is necessary to optimize the model for specific applications. The results indicate that our method provides a promising direction for real-time motion detection systems that require high precision and adaptability to varying conditions.

## Introduction

In the past decade, computer vision and video surveillance have undergone remarkable advancements, propelled by breakthroughs in deep learning, improved hardware capabilities, and the availability of vast datasets. These technologies have transitioned from traditional rule-based systems to more sophisticated and adaptable methods, enabling applications in diverse fields, including security, healthcare, and autonomous systems.

The emergence of deep learning, particularly convolutional neural networks (CNNs), has revolutionized computer vision tasks, allowing systems to learn intricate patterns and representations directly from data. This shift has significantly enhanced the accuracy and efficiency of object recognition, tracking, and segmentation in real-world scenarios.

Motion detection serves as a fundamental component in video surveillance, contributing to the identification of relevant events and reducing the volume of data to be processed. Several techniques have been employed for motion detection, each with its strengths and weaknesses:

One of the simplest techniques, frame differencing, involves subtracting consecutive frames to identify regions with significant pixel value changes. This technique is simple but may be sensitive to variations in lighting conditions. Traditional methods like ViBe (Visual Background Extractor) [1] use pixel-wise comparisons over time to distinguish moving objects from the static background. These techniques are computationally efficient but can struggle with dynamic scenes and gradual illumination changes.

Optical flow [2, 3] calculates the motion vectors of pixels between consecutive frames. While it provides accurate results for object movements, it can be sensitive to noise and challenges like occlusions. Statistical models, such as Gaussian Mixture Models (GMM) [4], represent the foreground and background pixel distributions. These models adapt to changing conditions but may struggle with complex scenes and dynamic backgrounds.

Despite the progress in motion detection techniques, challenges persist. Issues like illumination changes, shadows, and complex scene dynamics can impact the accuracy of detection. Researchers have addressed these challenges through innovative approaches. For instance, advanced background subtraction models [5, 6], ensemble methods [7, 8], and hybrid techniques combining multiple detection strategies have been proposed to enhance robustness and adaptability.

Dudu Guo et al. [9] propose a method for detecting moving vehicles in satellite videos based on an improved ViBe algorithm, aiming to improve moving vehicle detection accuracy and reduce missed and false detection rates. Their approach employs the semantic segmentation model DeeplabV3+ to extract relevant regions, significantly improving detection performance compared to conventional methods. However, their technique struggles with accurately extracting small objects and is not suitable for real-time applications due to high detection times.

Existing methods often fail to provide a dynamic model that is both computationally efficient and highly accurate for real-time applications. Building on the work of Dudu Guo et al., this paper introduces a novel solution that integrates an optimized semantic segmentation model into ViBe background subtraction. Our approach aims to refine the discrimination between moving objects and dynamic backgrounds by leveraging deep learning-based semantic segmentation. This enhances the accuracy and reliability of motion detection in scenarios with varying lighting conditions, moving elements, and complex scenes.

The contributions of this paper are as follows:

- Proposal of a novel U-Net based segmentation model incorporating the Squeeze and Excitation model.
- Integration of the new segmentation model into ViBe for enhanced motion detection performance.
- Implementation of adaptive techniques to enhance the technique’s robustness in complex scenarios.

The subsequent sections detail the methodology, experimental setup, and results of our proposed approach, demonstrating its potential advantages in real-world video surveillance applications.

## Related work

Motion detection is a fundamental task in computer vision, playing a vital role in various applications such as surveillance systems, human activity recognition, object tracking, gesture recognition, traffic monitoring, and autonomous vehicles. The aim is to determine how an object’s position changes with respect to its surroundings or how the surroundings change in relation to the object. However, challenges such as dynamic backgrounds, camera motion, shadow and illumination changes, inter-object occlusion, and abrupt motion make achieving accurate object detection extremely challenging. As a result, various algorithms have been developed, each with its strengths and shortcomings.

Background subtraction methods have gained widespread popularity due to their exceptional computational efficiency and effectiveness. However, the accuracy of the background model profoundly influences the outcomes of background subtraction-based detection. Hence, establishing a robust background model holds paramount importance in this approach. Considering the unique demands of video surveillance and the intricacies of surveillance environments, ViBe (Visual Background Extractor) emerges as an optimal choice. ViBe, initially introduced by O. Barnich et al. in 2011, stands out as a potent and efficient technique for background subtraction in video sequences.

This algorithm operates on a sample-based principle for detecting moving objects, offering advantages such as reduced computational overhead, minimal memory footprint, and rapid processing capabilities. These attributes render ViBe well-suited for real-time moving object detection.

Following the foundational work by O. Barnich et al., the ViBe technique has seen numerous refinements and adaptations to address specific challenges and broaden its applicability. Talab et al. [10] introduced an innovative approach for crack detection in videos, leveraging the ViBe algorithm in conjunction with multiple filtering methods. This extension demonstrated the versatility of ViBe beyond traditional surveillance applications, showcasing its potential for specialized domains such as infrastructure inspection. Gao and Cheng [11] applied the ViBe algorithm to extract smoke contours and shapes, leading to more accurate smoke detection. Their work highlighted the algorithm’s adaptability for environmental monitoring and safety applications, particularly in scenarios where smoke detection is crucial.

Nonetheless, certain limitations persisted within the general ViBe algorithm. Firstly, when the initial frame of a video includes a moving object, a lingering ’ghost area’ remains for an extended period before being effectively eliminated. Secondly, there is the issue of shadows. Addressing these concerns, Guangyi Tang et al. [12] made significant enhancements to the ViBe algorithm, introducing an advanced ViBe-based background subtraction technique. Their improvements primarily focused on more effective shadow removal and enhanced robustness in detecting moving objects, even in challenging and complex scenarios.

Further advancements were made by integrating deep learning models with ViBe to enhance its performance in complex environments. The combination of ViBe with convolutional neural networks (CNNs) has shown promise in improving the accuracy of object detection in challenging lighting conditions and cluttered backgrounds. Most notably, in 2023, Dudu Guo et al. proposed an enhanced version of the ViBe algorithm for moving vehicle detection in satellite videos. Recognizing the unique challenges posed by satellite imagery, such as large-scale movements, varying lighting conditions, and atmospheric effects, they employed the semantic segmentation model DeeplabV3+ to extract relevant regions and introduced adaptive learning rates for background model updates. This allowed the algorithm to better accommodate sudden changes in the environment. However, their technique struggles with accurately extracting small objects and is not suitable for real-time applications due to high detection times.

Despite the significant strides made, there remains much potential for further advancements, particularly in integrating advanced machine learning techniques for better adaptability and real-time performance. The existing techniques often struggle with balancing accuracy and computational efficiency, especially in complex and dynamic environments. Issues such as shadow removal, initial frame contamination, and handling small or occluded objects still pose significant challenges. These gaps have led to the design of the proposed methodology, which aims to leverage the strengths of both traditional background subtraction techniques and modern deep learning models to achieve more robust and real-time capable motion detection. By incorporating a Squeeze-and-Excitation (SE) block into the U-Net architecture, the proposed approach aims to enhance feature recalibration and improve overall detection accuracy, addressing the observed technical gaps in existing methods.

## Methodology

### A. Existing methods

#### 1. Motion detection (ViBe)

The ViBe algorithm operates on the principle of leveraging neighboring pixels to create a background model, which is then compared with the current pixel value to detect foreground elements. It consists of four main stages: background model initialization, foreground detection, foreground segmentation, and model updating.

*a) Background model initialization*. The background model is initialized using a single frame from the video sequence. This process involves randomly selecting neighboring pixels to serve as model sampling pixel values, taking into account the spatial distribution characteristics of adjacent pixels with similar values. The formula for initializing the background model is defined as follows:

$$M^{0}(x)=\{v^{0}[y|y\in N_{G}(x)]\}$$ (1)

Where *M*^0^(*x*) represents the initialized background model, *v*^0^(*y*) corresponds to the pixel value of the selected pixel in the initial video frame, *N*_*G*_(*x*) is the set of domain pixels associated with pixel point *x* in the first video frame, and *y* signifies the pixel value of the pixel chosen from within *N*_*G*_(*x*).

*b) Foreground detection*. Foreground detection is treated as a binary classification task, distinguishing between background and foreground pixels. Each pixel in the image corresponds to a sample set in the background model, which is then evaluated to determine if the pixel in the subsequent frame belongs to the foreground. The detection threshold is determined based on the Euclidean distance between the pixel value and the samples in the background model.


$$M(x)=\{v_{1},v_{2},\ldots ,v_{n}\}$$ (2)

Here *v*(*x*) is the pixel value at the pixel point *x* in the image, *M*(*x*) is the background model of the pixel point *x*, and *N* is the empirical value (taken as 20).

*c) Foreground segmentation*. A segmentation threshold is applied to classify pixel points as either background or foreground based on the number of background sample points that satisfy the threshold condition.

If we define the Euclidean distance between the pixel value *v*(*x*) and the sample of *M*(*x*) as:

$$dist\{v(x),v_{n}(x)\}$$ (3)

where *v*_*n*_(*x*)∈*M*(*x*) (*n* = 1,2,…*n*), and a foreground segmentation threshold R. If the number of background sample points satisfies the condition:

$$count\{dist\{v(x),v_{n}(x)\}>R\}\geq \beta$$ (4)

then the pixel point *M*(*x*) is classified as a background point, otherwise it is a foreground pixel.

ViBe is designed for efficient foreground object extraction in video sequences, making it suitable for real-time applications. Its adaptability to gradual scene changes and dynamic environments adds to its appeal. However, in challenging scenarios characterized by abrupt lighting changes or complex backgrounds with persistent patterns, ViBe may encounter difficulties, resulting in false positives or false negatives. To address these limitations, various extensions and modifications have been proposed, often involving the integration of additional motion detection techniques or the amalgamation with other computer vision methodologies.

#### 2. Segmentation (U-Net)

The U-Net architecture, introduced by Olaf Ronneberger et al. [13] has become a cornerstone in the field of image segmentation. U-Net is particularly acclaimed for its ability to accurately segment structures in biomedical images and has found widespread applications in various computer vision tasks.

The U-Net architecture is characterized by its U-shaped structure, which consists of a contracting path followed by an expansive path. The contracting path captures contextual information through convolutional and pooling layers, while the expansive path enables precise localization using transposed convolutions. Skip connections bridge the contracting and expansive paths, facilitating the transfer of high-resolution features to the final output.

The distinctive feature of U-Net is the incorporation of skip connections that concatenate feature maps from the contracting path to the corresponding layers in the expansive path. This enables the model to recover spatial information lost during down-sampling, contributing to more accurate segmentation. The is model trained by minimizing the pixel-wise binary cross-entropy loss [14]:

$$L(X,Y)=-\frac{1}{N}\sum_{i=1}^{N}\left[Y_{i}\cdot \log\left(P(X_{i})\right)+(1+Y_{i})\cdot \log\left(1-P(X_{i})\right)\right]$$ (5)

Where X denotes the input image, Y is the corresponding ground truth segmentation map and *P*(*X*_*i*_) is the predicted probability of the pixel *X*_*i*_ belonging to the foreground.

U-Net has demonstrated exceptional performance in various segmentation tasks, including biomedical image segmentation, object detection, and satellite image segmentation. Its versatility and efficacy stem from its ability to handle limited training data and its capacity to produce detailed segmentations.

#### 3. Squeeze and excitation

The Squeeze-and-Excitation (SE) model, proposed by Jie Hu et al. in [15] presents an innovative approach to enhance the representation power of neural networks by explicitly modeling channel-wise dependencies in feature maps. The SE model introduces a lightweight mechanism that adaptively recalibrates feature responses, thereby improving the discriminative capability of deep neural networks. Its elegance lies in its simplicity and efficiency, making it suitable for integration into various architectures across different computer vision tasks.

**Fig 1:**
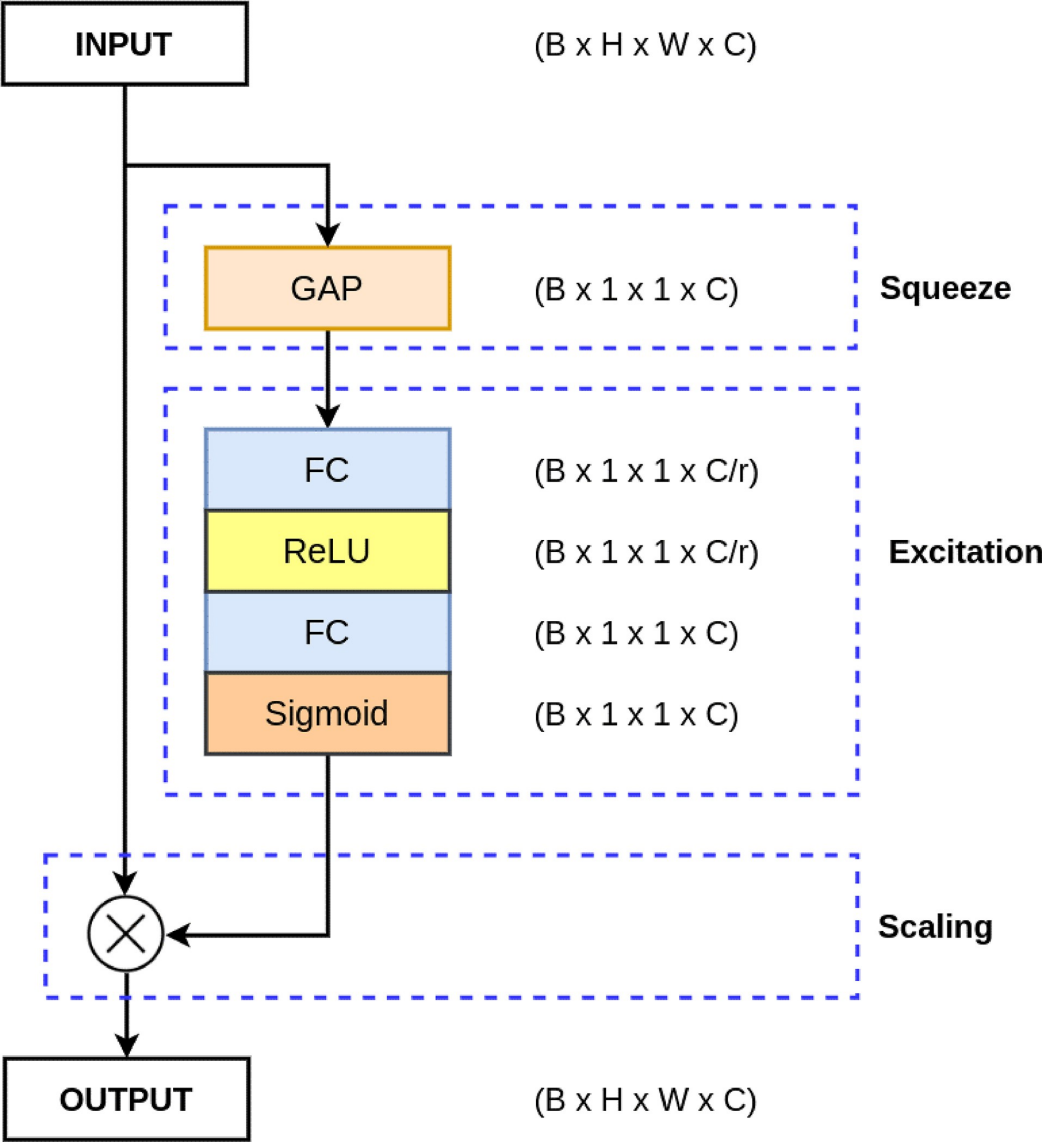
Squeeze and Excitation model architecture.

As shown in Fig 1, the SE model is composed of two key operations: squeezing and exciting. The squeezing operation involves global average pooling (GAP) [16] to condense feature maps into a channel descriptor. The exciting operation employs a set of fully connected layers to produce a set of channel-wise scaling factors. The combination of these operations forms the SE block, which is seamlessly integrated into existing neural network architectures.

The SE block operates as follows:

Let X represent the input feature maps of dimensions B×H×W×C, where B, H, W, and C denote batch size, height, width, and the number of channels, respectively.

Squeeze Operation:

$$z_{i}=\frac{1}{H\times W}\sum_{j=1}^{H}\sum_{k=1}^{W}x_{ijk}$$ (6)

Excitation Operation:

$$s_{i}=\sigma (W_{2}\delta (W_{1}z_{i}))$$ (7)

where *δ* is the ReLU activation function, *W*_1_ and *W*_2_ are learnable weights, and *σ* is the sigmoid activation function.

Scale Operation:

$${\hat{x}}_{ijk}=s_{i}\cdot x_{ijk}$$ (8)

SE models enhance accuracy and generalization across computer vision tasks like image classification, object detection, and semantic segmentation by enabling networks to focus on relevant features while suppressing irrelevant ones. They are lightweight and easily integrable into existing architectures, making them widely applicable in diverse deep learning frameworks and real-world applications.

### B. Proposed work

This study focuses on three key parts:

- A U-Net based segmentation model which makes use of a squeeze and excitation model to enhance performance is proposed.
- The enhanced segmentation model is integrated with ViBe to bring about better motion detection performance.
- Dynamic techniques are employed in the ViBe section to bolster robustness

#### 1. Enhanced segmentation

The proposed model is an instantiation of the Squeeze-and-Excitation U-Net (SE-UNet) model, combining the U-Net architecture with the Squeeze-and-Excitation (SE) block. This model is designed for semantic segmentation tasks, where the goal is to classify each pixel in an input image into predefined classes.

**Fig 2:**
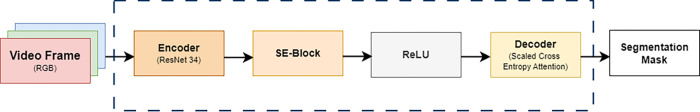
Proposed segmentation model architecture.

*a) Model architecture*. As shown in Fig 2. the core of SE-UNet is the U-Net architecture, known for its effectiveness in image segmentation. U-Net consists of a contracting path to capture context and an expansive path to enable precise localization, connected by skip connections to retain spatial information during down sampling.

The encoder is the ResNet34 [17] architecture. It extracts features from the input image through a series of convolutional and pooling layers. These extracted features are then passed to the Squeeze-and-Excitation Block.

The Squeeze-and-Excitation Block is a key component of the SE-Unet model. It computes a channel-wise attention score and scales the output of the encoder. The SE block enhances channel-wise dependencies, allowing the model to focus on informative channels and improve feature representations.

After passing through the SE Block, the features are sent to the Decoder. The Decoder uses Scaled Cross Entropy (SCSE) attention to combine low-resolution features from the encoder with high-resolution features from itself. This helps the model recover spatial information lost during the encoding process.

Finally, the output of the Decoder passes through the Rectified Linear Unit (ReLU) activation function [18]; however, this can be customized based on specific requirements. This produces the output segmentation mask which is used to segment the input image into different regions.

*b) Model initialization*. Encoder: The SEUnet employs a pre-trained ResNet34 encoder, which serves as the feature extractor. Other ResNet variants, such as resnet18 or resnet50, can be chosen based on specific requirements.

Encoder Weights: The encoder is initialized with weights pre-trained on the ImageNet dataset, facilitating the learning of meaningful hierarchical features.

Input and Output Channels: The model takes an input with a specified number of channels (in_channels) and produces an output with a specified number of classes (out_channels), typical for semantic segmentation tasks.

*c) Output*. The final output of the SE-UNet model is the segmented image with enhanced feature representations due to the combination of U-Net and Squeeze-and-Excitation.

The SE-UNet architecture benefits from both the contextual understanding of U-Net and the channel-wise attention mechanism of the SE block, making it well-suited for semantic segmentation tasks where capturing intricate details and dependencies is crucial.

#### 2. ViBe-segmentation integration

The segmentation (SE-UNet) model processes each frame to generate a semantic segmentation mask. Specifically, the model identifies regions corresponding to objects or foreground elements, producing a binary mask.

Simultaneously, the ViBe model is initialized with the first frame of the input video sequence. The background samples are generated using a predefined number of samples with the option to adapt the minimum required matches and a specified blur radius. To handle variations in motion and illumination, an adaptive blur kernel is employed based on the presence of motion in the initial frame. The ViBe model is iteratively updated for each subsequent frame in the video sequence.

To account for evolving background characteristics, the ViBe model is periodically updated with new background samples. The update is triggered at predefined intervals, and new background samples are extracted from regions not classified as foreground in the refined motion mask.

**Fig 3:**
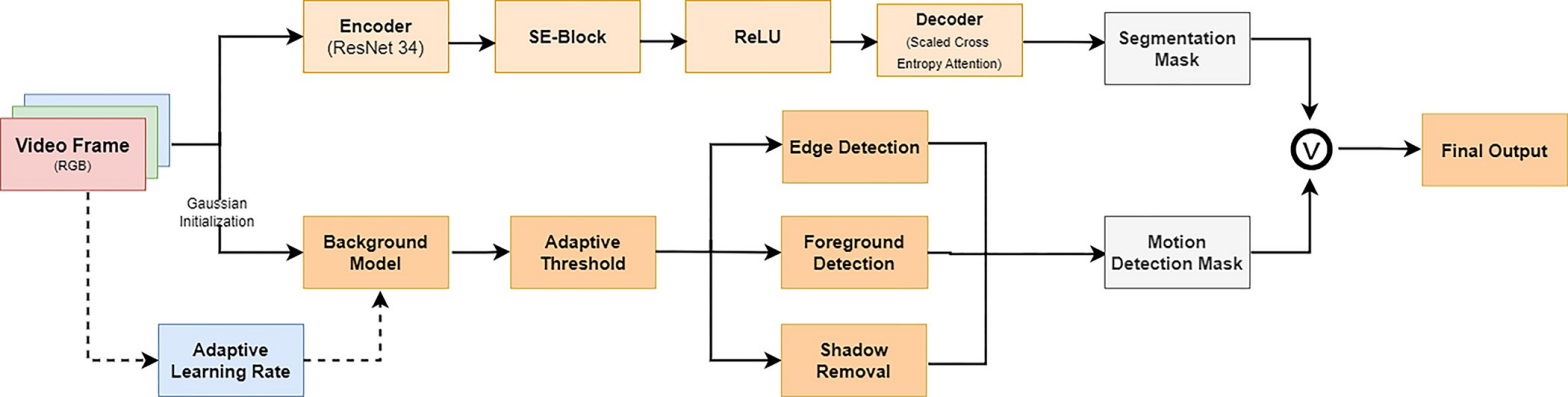
SE-ViBe architecture.

The binary motion mask obtained from ViBe and the semantic segmentation mask from SE-UNet are fused using a bitwise AND operation. This combined mask enhances the motion detection capability by incorporating semantic information from SE-UNet, improving the discrimination between moving objects and the background. The integration is illustrated in Fig 3:

#### 3. Dynamic detection

To adapt to varying scenarios, an adaptive learning rate mechanism is integrated into the ViBe model. The learning rate is dynamically adjusted based on the proportion of pixels indicating motion in the combined mask. This adaptive strategy ensures responsiveness to changes in the scene and contributes to the model’s robustness.

*a) Adaptive threshold*. The adaptive threshold, denoted as *min*_*matches*, plays a crucial role in determining the minimum number of matches necessary to classify a pixel as part of the background within the ViBe motion detection algorithm. Unlike a fixed threshold, the adaptive threshold dynamically adjusts based on the mean intensity of the current frame, calculated using the frame’s mean intensity, *frame*.*mean*(). This adaptive nature allows the algorithm to adapt to varying lighting conditions present in different frames.

A baseline value of 4 is used which ensures a minimum number of matches required for background classification, even in relatively bright scenes. As a result, the threshold becomes more stringent as the mean intensity of the image increases, thereby improving the accuracy of background detection across diverse environmental conditions.


$$\alpha =\frac{1}{motion\_mask.mean()+1}$$ (9)

This approach makes the algorithm robust to varying lighting conditions. In darker scenes, fewer matches are required to classify a pixel as background, and in brighter scenes, more matches are needed.

*b) Adaptive learning rate*. The adaptive learning rate (*α*) serves as a pivotal parameter within the ViBe motion detection algorithm, governing the rate at which the background model adjusts to variations in the scene, particularly in motion intensity. This parameter, *α*, operates in an inverse relationship with the intensity of motion observed in the scene.

Mathematically, the learning rate (*α*) is determined as the reciprocal of the motion intensity present in the scene, calculated as, *motion*_*mask*.*mean*() + 1. The addition of "+1" is essential to prevent division by zero. Consequently, a higher *α* signifies a swifter adaptation of the background model to changes in the scene, while a lower *α* results in a more gradual adjustment rate. It is given by the equation:

$$min\_matches=int\left(4+\frac{frame.mean()}{50}\right)$$ (10)

Here *motion*_*mask* is the binary mask indicating motion in the current frame and *motion*_*mask*.*mean*() calculates the mean value of the motion mask, representing the motion intensity. By dynamically adjusting *α* based on the motion intensity, the algorithm effectively optimizes the background modeling process to maintain accurate motion detection across varying environmental conditions.

#### 4. System model

In this section, we present the mathematical modeling of our proposed motion detection and segmentation technique. This model provides a precise and formal description of the underlying system, facilitating a deeper understanding of its operational principles and performance characteristics.

The proposed system integrates a ViBe-based background subtraction method with a U-Net-based semantic segmentation model. The system is designed to detect and segment moving objects in video sequences. Below are the key components and their mathematical representations:

1. Initialization of the Background Model:

Let *I*_*t*_ denote the input frame at time *t*. The initial background model *B* is initialized using the first frame *I*_0_:

$$B=I_{0}$$ (11)

The ViBe algorithm maintains multiple background samples for each pixel. Let *S*(*x*,*y*) be the set of samples for the pixel at position (*x*,*y*):

$$S(x,y)=\{B(x,y),B_{1}(x,y),B_{2}(x,y),\ldots ,B_{N-1}(x,y)\}$$ (12)

where *N* is the number of samples (48 in this implementation).

2. Foreground Detection with ViBe:

For each pixel *p* in the frame *I*_*t*_, the difference *D*(*p*) with the samples in *S*(*p*) is computed:

$$D(p,S(p))=\underset{s\in S(p)}{\lim}\left\|I_{t}(p)-s\right\|$$ (13)

If the minimum distance *D*(*p*, *S*(*p*)) is greater than a threshold *θ*, the pixel is classified as foreground:

$$F_{t}(p)=\left\{ \begin{array}{l} 1\;if\;D(p,S(p))>\theta \\ 0\;otherwise \end{array}\right.$$ (14)

where *F*_*t*_(*p*) is the foreground mask.

3. Adaptive Thresholding:

The threshold *θ* is dynamically adjusted based on the mean intensity of the frame:

$$\theta =\theta_{0}+k.mean(I_{t})$$ (15)

where *θ*_0_ is the base threshold and *k* is a scaling factor.

4. SE-UNet Based Segmentation:

The SE-UNet model takes the input frame *I*_*t*_ and produces a segmentation mask *M*_*t*_:

$$M_{t}=SEUNet(I_{t})$$ (16)

The SE-UNet model is trained to output a probability map indicating the likelihood of each pixel belonging to a moving object. The binary mask is obtained by thresholding this probability map:

$$M_{t}(p)=\left\{ \begin{array}{l} 1\;if\;SEUNet(I_{t}(p))>0.5\\ \;0\;otherwise \end{array}\right.$$ (17)

5. Combining Masks:

The final foreground mask $F_{t}^{\prime }$ is obtained by combining the ViBe and SE-UNet masks using a logical OR operation:

$$F_{t}^{\prime }=F_{t}(p)\vee M_{t}(p)$$ (18)

6. Background Model Update:

The background model is updated using the current frame *I*_*t*_ and the combined foreground mask $F_{t}^{\prime }$:

$$B_{t+1}(p)=\left\{ \begin{array}{l} I_{t}(p)\;if\;F_{t}^{\prime }(p)=0\\ B_{t}(p)\;otherwise \end{array}\right.$$ (19)

The pseudo code is detailed below:

*# Initialization*

*ViBeWithSegmentation.initialize(first_frame)*

*# Main Loop*

*for each frame in video:*

 *# Convert the frame to grayscale*

 *gray_frame = convert_to_grayscale(frame)*

 *# Segmentation*

 *segmentation_mask = ViBeWithSegmentation.get_segmentation_mask(frame)*

 *# Input: frame (RGB), Output: segmentation_mask (binary)*

 *# Background Subtraction (ViBe)*

 *vibe_foreground_mask = ViBeWithSegmentation.update(gray_frame)*

 *# Input: frame (grayscale), Output: vibe_foreground_mask (binary)*

 *# Adaptive Learning Rate (optional if used inside ViBe model)*

 *learning_rate = ViBeWithSegmentation.adaptive_learning_rate(vibe_foreground_mask)*

 *# Input: vibe_foreground_mask (binary), Output: learning_rate (float)*

 *# Updating ViBe Model with New Samples*

 *new_samples = get_new_background_samples(gray_frame, vibe_foreground_mask)*

 *ViBeWithSegmentation.update_with_new_samples(new_samples)*

 *# Inputs: new_samples (grayscale), vibe_foreground_mask (binary)*

 *# Combine masks*

 *combined_mask = combine_masks(vibe_foreground_mask, segmentation_mask)*

 *# Inputs: vibe_foreground_mask (binary), segmentation_mask (binary), Output: combined_mask (binary)*

 *# Result—Motion Detected Frame*

 *result_frame = apply_mask_to_frame(frame, combined_mask)*

 *# Inputs: frame (RGB), combined_mask (binary), Output: result_frame (RGB)*

 *# Output or further processing with result_frame*

 *end for*

#### 5. Model training

*a) Dataset*. For training and validation, we utilized the ChangeDetection.net 2012 dataset [19], which encompasses a diverse range of scenarios including dynamic backgrounds, illumination changes, and shadow effects. This dataset is widely used in the motion detection and computer vision community, providing a robust benchmark for evaluating algorithm performance.

*b) Data preprocessing*. Data preprocessing involved several steps to prepare the input frames for training. Firstly, the frames were resized to a resolution of 256x256 pixels to standardize input dimensions and reduce computational complexity. Each frame was then normalized to have pixel values in the range [0, 1].

Secondly, to improve the generalization capability of the model, several data augmentation techniques were applied to the training data:

1. Horizontal Flipping: Frames were randomly flipped horizontally with a probability of 0.5.
2. Random Cropping: Random crops of size 224x224 pixels were extracted from the resized frames.
3. Rotation: Frames were randomly rotated within a range of -10 to +10 degrees.

These augmentations helped the model learn invariant features and enhanced its performance on unseen data.

*c) Training schedule*. The SE-ViBe model was trained for 50 epochs using a batch size of 16. The Adam optimizer, with an initial learning rate of 0.001, was employed due to its adaptive learning rate properties, facilitating efficient convergence. The learning rate was decayed by a factor of 0.1 every 10 epochs to fine-tune the model as training progressed. Binary cross-entropy loss was chosen for its advantages in segmentation tasks; it measured pixel-wise classification accuracy and ensured a strong overlap between predicted and ground truth masks. Additionally, binary cross-entropy encouraged the model to make confident predictions by penalizing less certain classifications, thus enhancing the overall performance and robustness of the segmentation model.

#### d) Regularization techniques

To prevent overfitting and improve generalization, the following regularization techniques were employed:

1. Dropout: A dropout rate of 0.5 was applied to the fully connected layers in the SE modules to randomly deactivate neurons during training, promoting redundancy in the feature representations.
2. Weight Decay: L2 regularization with a coefficient of 0.0001 was applied to the weights of the convolutional layers to penalize large weights and encourage simpler models.

By employing these preprocessing, augmentation, and regularization strategies, the SE-ViBe model was trained to achieve high accuracy in detecting moving objects in video streams while maintaining robustness across various challenging scenarios.

## Experiments

To evaluate the efficacy of our proposed method, we conducted experiments using benchmark datasets from changedetection.net 2012 dataset, which covers six distinct categories: baseline, camera jitter, dynamic background, intermittent object motion, shadow, and thermal. In our investigation, we focused on five benchmark datasets: Boats, Bungalows, Fall, Highway, and Pedestrians. We employed seven key metrics to assess detection performance: recognition rate of background (SP), recognition rate of foreground (RE), false positive rate (FPR), false negative rate (FNR), percentage of wrong classification (PWC), precision (PRE), and F-score (F). Higher values for RE, SP, PRE, and F indicate more accurate detection of the target area, while lower values for FPR, FNR, and PWC suggest more precise background detection. The hardware configuration for the experiment consisted of 125.5G RAM, i9-10900X CPU, 2.60GHz, RTX3090 graphics card,, Ubuntu 20.40 development environment,OpenCV4.5.6, and Python3.7.

### A. Overall analysis

**Fig 4:**
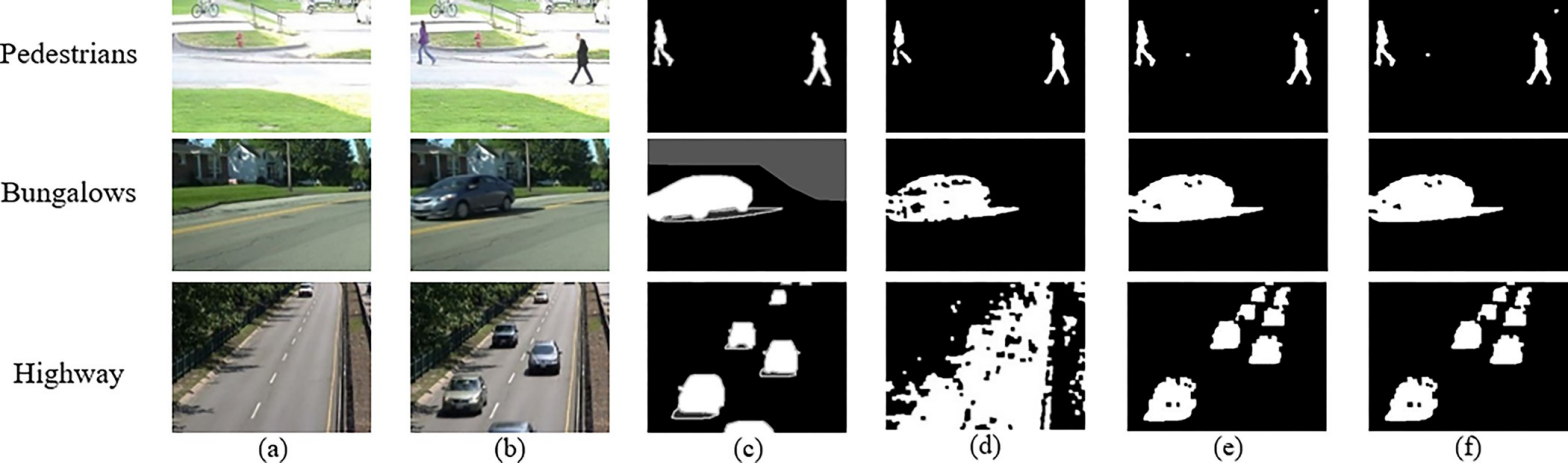
The motion detection results on the videos ‘Bungalows’ and ‘Pedestrians’: (a) first frame; (b) frame for detection; (c) ground-truth; (d) G-ViBe result; (e) D3-ViBe result; (f) SE-ViBe result.

Our proposed SE-ViBe technique was compared against the original ViBe approach (G-ViBe) and a DeeplabV3+ based model (D3-ViBe)—loosely based on the method proposed in [9]. Under stable conditions, particularly observed in the Bungalows dataset, all three techniques demonstrate comparable performance levels, with SE-ViBe and D3-ViBe showing approximately 7.8% and 6.9% improvement respectively for the F1 score when compared to G-Vibe. Also, SE-ViBe exhibits better consistency over G-ViBe and D3-ViBe.The experimental results are detailed in Table 1 and Fig 4 below:

**Table 1:** Performance metrics for G-ViBe, D3-ViBe and SE-ViBe for ‘Pedestrians’, ‘Bungalows’ and ‘Highway’.

Performance Metrics	Pedestrians			Bungalows			Highway		
	G-ViBe	D3-ViBe	SE-ViBe	G-ViBe	D3-ViBe	SE-ViBe	G-ViBe	D3-ViBe	SE-ViBe
SP	**0.9993**	0.9986	0.9979	0.9761	**0.9789**	0.9612	0.9414	**0.9853**	0.9820
RE	0.9031	0.9661	**0.9712**	0.8742	0.9356	**0.9534**	0.8333	0.9436	**0.9500**
FPR	**0.0007**	0.0014	0.0021	0.0238	**0.0211**	0.0388	0.0585	**0.0147**	0.0180
FNR	0.0968	0.0339	**0.0288**	0.1257	0.0644	**0.0466**	0.1667	0.0564	**0.0500**
PWC	**0.0015**	0.0016	0.0024	0.0294	**0.0235**	0.0396	0.0641	**0.0169**	0.0201
PRE	**0.9095**	0.8517	0.8519	0.6811	**0.7274**	**0.7274**	0.4356	**0.7854**	0.7853
F	0.9063	0.9053	**0.9076**	0.7657	0.8184	**0.8252**	0.5721	0.8573	**0.8599**

A more detailed examination of the different techniques reveals distinct strengths favoring SE-ViBe. This distinction becomes particularly evident when analyzing performance metrics on datasets characterized by challenging conditions and the presence of shadows, such as the Highway dataset. The Highway dataset is prone to abrupt variations in lighting. In these complex scenarios, the robustness of SE-ViBe becomes apparent, as shown in Table 1. SE-ViBe outperforms G-ViBe in every metric, notably giving a 50.3% increase in the F1 score when compared to G-ViBe and performs just as well as D3-ViBe but with significantly faster detection times (as discussed in the subsection “Discussion”).

### B. Ablation study

This section comprises two parts. Firstly, we explore the impact of integrating the squeeze and excitation model into U-Net, and secondly, we analyze how adaptive techniques influence the model’s performance.

#### 1. Squeeze and excitation

To assess the impact of the Squeeze and Excitation (SE) model on our motion detection framework, SE-ViBe, we conducted an ablation study aimed at evaluating its contribution. The study involved removing the SE model from the UNet architecture and analyzing its effect on detection performance. The results are presented in Tables 2 and 3 below:

**Table 2:** Performance metrics for G-ViBe, D3-ViBe and SE-ViBe for the datasets Pedestrians and Bungalows.

Performance Metrics	Pedestrians		Bungalows	
	SE-ViBe	UNet-ViBe	SE-ViBe	UNet-ViBe
SP	0.9612	**0.9780**	0.9820	**0.9853**
RE	**0.9534**	0.9376	**0.9500**	0.9437
FPR	0.0388	**0.0220**	0.0180	**0.0147**
FNR	**0.0466**	0.0624	**0.0500**	0.0563
PWC	0.0396	**0.0244**	0.0201	**0.0169**
PRE	**0.7274**	0.7274	0.7853	**0.7855**
F	**0.8252**	0.8192	**0.8599**	0.8573

**Table 3:** Performance metrics for G-ViBe, D3-ViBe and SE-ViBe for the Fall and Canoe.

Performance Metrics	Fall		Canoe	
	SE-ViBe	UNet-ViBe	SE-ViBe	UNet-ViBe
SP	0.9564	**0.9590**	0.9710	**0.9821**
RE	**0.8084**	0.8003	**0.7088**	0.6163
FPR	0.0436	**0.0410**	0.0290	**0.0179**
FNR	**0.1916**	0.1997	**0.2912**	0.3837
PWC	0.0463	**0.0437**	0.0398	**0.0287**
PRE	**0.2510**	0.2507	**0.5127**	0.5113
F	**0.3830**	0.3818	**0.5950**	0.5589

While both architectures achieved comparable performance levels in the initial analysis, as presented in Table 2, SE-UNet demonstrated greater adaptability when confronted with complex scenes, as shown in Table 3. In datasets such as ’Fall’ and ’Canoe’, characterized by dynamic backgrounds due to factors like shaking leaves and water movement, SE-UNet exhibited heightened robustness and generalization capabilities. Notably, SE-UNet showcased a reduction in both false positives and false negatives, further underscoring its superiority. The integration of SE modules introduces a valuable layer of adaptability and context-aware feature refinement, positioning the SE-UNet as a promising architecture for a diverse range of computer vision tasks.

#### 2. Adaptive methods

The ablation study on adaptive learning rate and adaptive threshold techniques revealed their significant impact on the model’s performance in motion detection, as shown in Table 4. Comparing fixed values for these parameters against their adaptive counterparts, we observed notable differences. Specifically, using a fixed learning rate instead of an adaptive one resulted in a substantial increase in false positives, indicating a higher rate of incorrect background classifications. This was clearly demonstrated on the Pedestrians dataset, where the precision and F1 score dropped by 85% and 75%, respectively, with a fixed learning rate. Similarly, employing a fixed threshold instead of an adaptive one also led to elevated false positives, compromising the model’s ability to accurately distinguish between foreground and background elements, and resulting in a noticeable decrease in overall accuracy.

**Table 4:** Ablation study on the effects of adaptive learning rate and the adaptive thresholding on overall performance.

Performance Metrics	Bungalows			Highway			Pedestrians		
	SE-ViBe	Fixed LR	Fixed Threshold	SE-ViBe	Fixed LR	Fixed Threshold	SE-ViBe	Fixed LR	Fixed Threshold
SP	**0.9612**	0.9332	0.9559	**0.9820**	0.6517	0.9404	**0.9979**	0.9178	0.9974
RE	0.9534	**0.9707**	0.9688	0.9500	**0.9818**	0.9767	0.9712	**0.9849**	0.9740
FPR	**0.0388**	0.0668	0.0441	**0.0180**	0.3483	0.0595	**0.0021**	0.0822	0.0026
FNR	0.0466	**0.0293**	0.0312	0.0500	**0.0182**	0.0233	0.0288	**0.0151**	0.0260
PWC	**0.0396**	0.0636	0.0429	**0.0201**	0.3252	0.0568	**0.0024**	0.0814	0.0029
PRE	**0.7274**	0.5807	0.6971	**0.7853**	0.1750	0.5692	**0.8519**	0.1255	0.8125
F	**0.8252**	0.7267	0.8107	**0.8599**	0.297	0.7193	**0.9076**	0.2226	0.8860

* Fixed Threshold: num_samples = 10; Fixed Learning Rate = 0.01

These findings underscore the critical role of an adaptive learning rate in enhancing system performance, while also highlighting the significant contributions of adaptive thresholding in improving model accuracy. Furthermore, the study illustrates the interdependency between the two techniques in maintaining high performance. While both adaptive learning rate and adaptive thresholding are crucial for optimizing model performance, the adaptive learning rate emerges as the primary determinant of system efficacy in motion detection applications.

## Discussion

The results presented in the ‘Experiments’ section underscore the effectiveness of the SE-ViBe motion detection approach, which builds upon the ViBe framework, in effectively managing complex scenarios, outperforming both G-ViBe and D3-ViBe. This section delves into the computational complexity and memory requirements of the proposed algorithm and examines its limitations.

### 1. Computational complexity

The time complexity of G-ViBe can be broken down into three stages: background model initialization, background model update, and morphological operations. Among these stages, the background model update is the most significant contributor to the overall time complexity, with a complexity of *O*(*height*×*width*×*num*_*samples*). Other operations have a comparatively lower impact on the overall complexity.

As shown in the Table 5, SE-ViBe, on average, takes approximately 159% longer than G-ViBe. This increase in time can be attributed to the incorporation of additional layers for deep learning, plus adaptive learning rate computation and periodic background model updates. Despite this substantial increase in processing time, SE-ViBe still maintains sufficiently high speeds for real-time performance in many applications. Further research is necessary to optimize detection times and bring them more in line with the performance of G-ViBe.

**Table 5:** Real-time performance comparison of the three techniques on ’Boats’ and ’Fall’ datasets.

	Time(s)		
	G-ViBe	D3-ViBe	SE-ViBe
**Boats** (320*240)	144.993	1634.805	662.332
**Fall** (720*480)	368.238	1138.682	667.278

Conversely, D3-ViBe exhibits the slowest performance among the compared algorithms, taking on average 108.6% longer than SE-ViBe (440.5% longer than G-ViBe). This extended detection time renders D3-ViBe unsuitable for real-time applications. The significant increase is primarily due to the more complex model architecture of DeepLabv3+, which underpins D3-ViBe. The complexity of DeepLabv3+ includes multiple atrous convolutions and a larger number of parameters, resulting in longer processing times that make it unsuitable for real-time applications. In contrast, SE-ViBe, based on the simpler U-Net architecture, achieves a better balance between computational complexity and detection accuracy.

### 2. Memory requirements

The memory requirements of the SE-ViBe algorithm are influenced by several factors, including the storage of the background model, intermediate feature maps, and model parameters. The background model requires memory proportional to

*O*(*height*×*width*×*num*_*samples*), where each pixel stores multiple samples for background subtraction.

The integration of SE modules within the U-Net architecture also affects memory usage. SE modules introduce additional parameters and intermediate feature maps, which increase the memory footprint. However, the use of global average pooling within SE modules helps to mitigate the impact on memory by reducing the size of intermediate activations.

Compared to D3-ViBe, which is based on the DeepLabv3+ architecture, SE-ViBe has a relatively lower memory requirement. DeepLabv3+ employs multiple atrous convolutions with different rates, resulting in a significant number of intermediate feature maps and parameters that need to be stored in memory. This leads to a higher memory footprint for D3-ViBe.

Overall, SE-ViBe presents a trade-off between computational complexity and memory requirements, achieving a balance that allows for real-time performance while maintaining high detection accuracy. Further optimizations could focus on reducing the computational overhead and memory usage of SE modules, as well as exploring more efficient background model update strategies to enhance runtime performance.

### 3. Limitations

**Fig 5:**
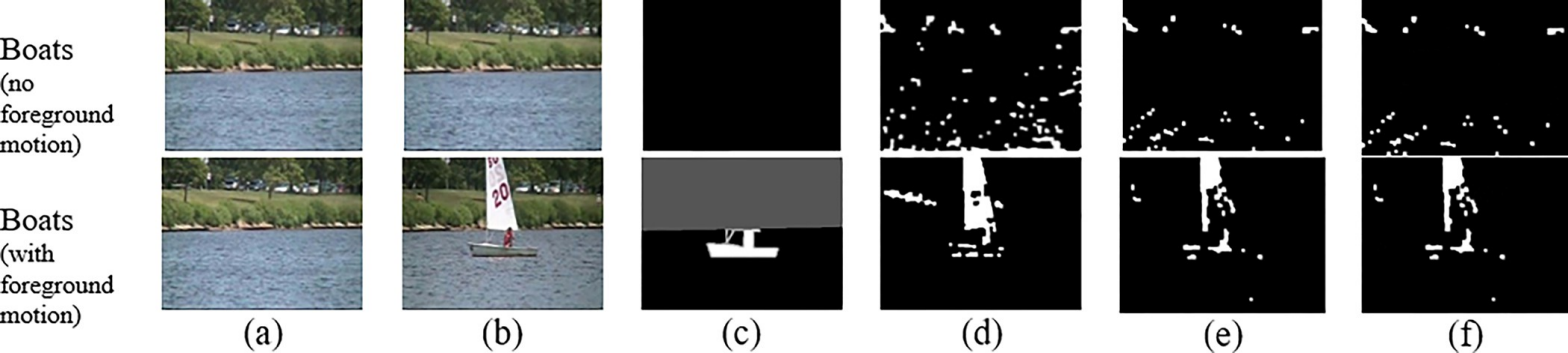
The motion detection results on ‘Boats’: (a) first frame; (b) frame for detection; (c) ground-truth; (d) G-ViBe result; (e) D3-ViBe; (f) SE-ViBe result.

It is important to acknowledge that SE-ViBe has demonstrated sub-optimal performance in certain scenarios. Table 6 and Fig 5 present the outcomes of experiments conducted on the Boats dataset, where SE-ViBe yielded unsatisfactory results. G-ViBe produced slightly better results, with D3-ViBe exhibiting the worst performance. This could potentially be attributed to limitations in the training data, which may negatively affect the effectiveness of the SE-ViBe model. The ‘Boats’ dataset exposes the segmentation model to data on which it does not generalize well, warranting further investigation to gain a deeper understanding of this issue. Despite these limitations, it is worth noting that the model has exhibited the capability to outperform even state-of-the-art models in many contexts.

**Table 6:** Performance metrics for G-ViBe, D3-ViBe and SE-ViBe on “Boats” dataset.

Performance Metrics	Boats		
	G-ViBe	D3-ViBe	SE-ViBe
SP	**0.9999**	0.9997	0.9983
RE	0.2777	0.1083	**0.4234**
FPR	**0.0001**	0.0003	0.0017
FNR	0.7224	0.8917	**0.5766**
PWC	**0.0038**	0.0058	0.0058
PRE	**0.9103**	0.7227	0.6439
F	0.4255	0.1884	**0.5109**

Additionally, SE-ViBe’s performance in detecting small objects is limited. The model’s segmentation capability is not finely tuned to accurately capture very small moving objects, leading to missed detections. Another limitation is the presence of ’ghost’ areas when initial frames contain moving objects, which persist until the model adapts. However, this problem is typically resolved in subsequent frames.

These limitations highlight areas for further research and improvement. Enhancing small object detection may require additional fine-tuning of the segmentation model. Reducing computational overhead without compromising accuracy is crucial for making SE-ViBe suitable for real-time applications. This can include optimizing the deep learning model through pruning and quantization, and employing adaptive methods that adjust model complexity based on scene requirements. The work by Dudu Guo et al. on the scale-center distance constraint can be explored to further boost detection performance. Expanding training and testing across diverse datasets will also help fine-tune the model’s robustness and generalizability, leading to a more effective motion detection system.

## Conclusion

This paper presents a novel segmentation model built upon the U-Net architecture, enhanced with the integration of the squeeze and excitation mechanism aimed at bolstering performance. The incorporation of the SE-UNet model into the ViBe motion detection technique yields noteworthy improvements in detection accuracy and performance. Notably, the utilization of dynamic tools plays a pivotal role in fortifying the model’s robustness, thereby improving its effectiveness without necessitating extensive parameter tuning. Moreover, an observed decrease in false positives underscores the model’s improved precision. Additionally, SE-ViBe runs 52.1% faster than D3-ViBe while showcasing better detection performance, making it more suitable for real-time applications. However, further training of the segmentation model is essential to fine-tune it for specific tasks, ensuring the attainment of optimal results.
